# Long-Term Effects of Financial Incentives for General Practitioners on Quality Indicators in the Treatment of Patients With Diabetes Mellitus in Primary Care—A Follow-Up Analysis of a Cluster Randomized Parallel Controlled Trial

**DOI:** 10.3389/fmed.2021.664510

**Published:** 2021-10-26

**Authors:** Rahel Meier, Corinne Chmiel, Fabio Valeri, Leander Muheim, Oliver Senn, Thomas Rosemann

**Affiliations:** Institute of Primary Care, University of Zurich and University Hospital Zurich, Zurich, Switzerland

**Keywords:** quality indicators—healthcare, hypertension management, diabetes management and control, pay for performance (P4P), financial incentive, primary care, chronic conditions and diseases

## Abstract

**Background:** The effect of financial incentives on the quality of primary care is of high interest, and so is its sustainability after financial incentives are withdrawn.

**Objective:** To assess both long-term effects and sustainability of financial incentives for general practitioners (GPs) in the treatment of patients with diabetes mellitus based on quality indicators (QIs) calculated from routine data from electronic medical records.

**Design/Participants:** Randomized controlled trial using routine data from electronic medical records of patients with diabetes mellitus of Swiss GPs.

**Intervention:** During the study period of 24 months, all GPs received bimonthly feedback reports with information on their actual treatment as reflected in QIs. In the intervention group, the reports were combined with financial incentives for quality improvement. The incentive was stopped after 12 months.

**Measurements:** Proportion of patients meeting the process QI of annual HbA1c measurements and the clinical QI of blood pressure levels below 140/85 mmHg.

**Results:** A total of 71 GPs from 43 different practices were included along with 3,854 of their patients with diabetes mellitus. Throughout the study, the proportion of patients with annual HbA1c measurements was stable in the intervention group (78.8–78.9%) and decreased slightly in the control group (81.5–80.2%) [odds ratio (OR): 1.21; 95% CI: 1.04–1.42, *p* < 0.05]. The proportion of patients achieving blood pressure levels below 140/85 mmHg decreased in the control group (51.2–47.2%) and increased in the intervention group (49.7–51.9%) (OR: 1.18; 95% CI: 1.04–1.35, *p* < 0.05) where it peaked at 54.9% after 18 months and decreased steadily over the last 6 months.

**Conclusion:** After the withdrawal of financial incentives for the GPs after 12 months, some QIs still improved, indicating that 1 year might be too short to observe the full effect of such interventions. The decrease in QI achievement rates after 18 months suggests that the positive effects of time-limited financial incentives eventually wane.

## Introduction

Improving the quality of care for the chronically ill, especially in patients with diabetes mellitus, is an important issue in all industrialized countries (1). At the level of healthcare providers, various strategies such as audit and feedback, clinical education, clinical reminders, or financial incentives have been pursued to narrow the gap between actual and optimal care (2). Financial incentives for healthcare providers, also called pay for performance (P4P) strategies when combined with quality indicators (QIs), have been studied in different settings. However, evidence concerning their effect on the quality of care, especially in ambulatory settings, is still inconclusive and little information is available from randomized controlled trials.

Not only the immediate effect of financial incentives is of interest but also further developments after their removal. Financial incentives often diminish when performance approaches the achievable maximum or when the financial resources are directed to quality-improvement efforts in other clinical areas. With limited resources available, the sustainability of improvements in quality of care is crucial for the long-term effectiveness of financial incentives. Former analyses in the United Kingdom and the United States of America showed conflicting evidence, with results ranging from declining performance to sustained performance levels (3–7).

In Switzerland, no real-life data on the P4P approach exists, and the use of QIs, especially in primary care, has been marginal. With this cluster-randomized parallel controlled trial, we aimed to test whether financial incentives are more effective than evidence-based educational feedback reports in the increasing proportion of patients with diabetes mellitus meeting specific QIs. Results directly after the intervention phase (12 months) indicated a little effect on directly incentivized QIs (8). With this analysis, we aimed to assess the long-term effects and sustainability of financial incentives after their removal.

## Methods

### Study Design and Participants

We conducted a follow-up analysis of a cluster-randomized parallel controlled trial in Swiss primary care using data from the FIRE database (family medicine international classification of primary care (ICPC) research using electronic medical records) (9). General practitioners (GPs) who participate in the FIRE project periodically contribute anonymized data from their electronic medical records (EMRs) to the steadily growing database holding the following components: administrative information, vital signs, laboratory values, medication data, and diagnostic codes according to the ICPC-2 classification scheme (10). At the start of recruitment in June 2018, more than 400 GPs participated in the FIRE project, and records from more than 500,000 patients and 5 million consultations were available.

**Figure 1:**
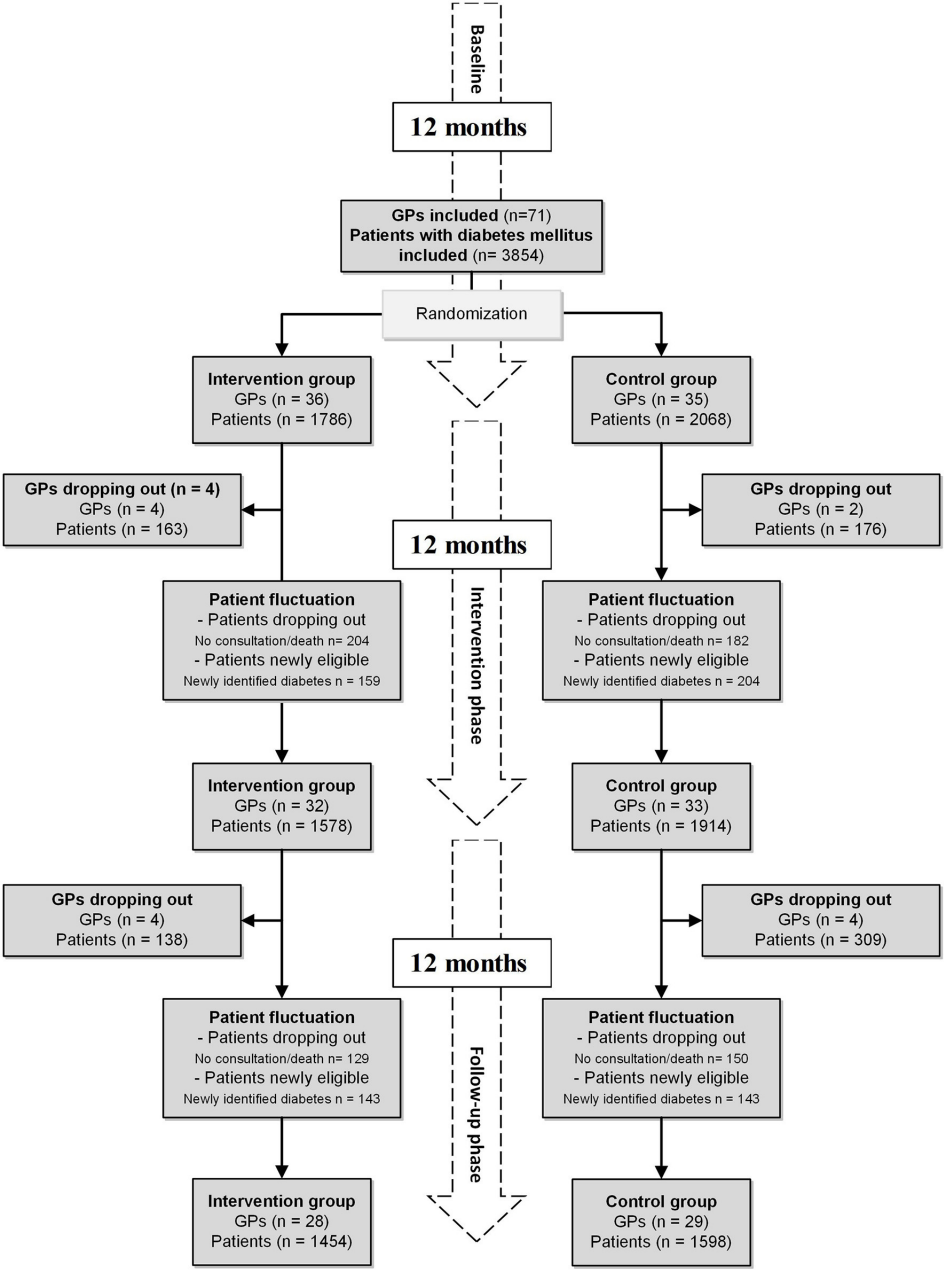
Flowchart of the study, including dropouts of GPs and patient fluctuation. Financial incentives were stopped after the intervention phase. GP, general practitioner.

In June 2018, all eligible GPs received an invitation to participate in the study [for the detailed eligibility criteria regarding data availability and data quality, see Meier et al. (8)]. We included all patients listed in the EMRs from all participating GPs with a diagnosis of diabetes mellitus who met at least one of the following criteria: (1) ICPC-2-code T89 (insulin-dependent diabetes mellitus) or T90 (insulin-independent diabetes mellitus). (2) Antidiabetic medication according to the anatomical therapeutic chemical classification system (A10A, A10B, and A10X) (11). Patients with diabetes mellitus were only eligible if they were diagnosed at least 4 months before the QI assessment and had at least one consultation within the last 12 months. During the study period, patients with newly identified diabetes mellitus were included, whereas patients without consultation within the last 12 months or reported dead were excluded from the analysis. For the flowchart of the study, see Figure 1.

According to the local Ethics Committee of the Canton of Zurich, the project did not fall under the scope of the law on human research and therefore did not require ethical approval (BASEC-Nr. Req-2017-00797). The trial was registered in the ISRCTN registry (identifier: ISRCTN13305645), and the study protocol has been published (12).

### Intervention

During the intervention and follow-up phase, GPs in both study groups received bimonthly diabetes feedback reports, containing information on their patients with diabetes mellitus (age, gender, and body mass index), the proportion of patients with at least one HbA1c measurement within the previous 12 months, and the proportion of patients with blood pressure measurements and achieving the target blood pressure level. The reports were generated from each participating GP's own EMR data to provide an individualized overview of his performance. In their key messages, the reports addressed various issues in the treatment of patients with diabetes mellitus, such as the management of cardiovascular risk or the prevention of disease-related complications. An example of the report can be found in Supplementary Material 1. The intervention group was informed, after randomization, that they would receive a financial incentive of 75 Swiss francs per percentage point improvement in the reported QIs after the intervention period (see Table 1 for incentivized QI description). Simulations beforehand had shown that this incentive could add up to a significant financial amount for the GPs concerning their incomes. The control group was blinded for the financial incentive of the intervention group. The financial incentive was the only difference between the two groups.

**Table 1:** Quality indicators are used to assess primary and secondary outcomes.

**Type**	**Subject**	**Description**
**Incentivized QIs**		
Clinical QI	Blood pressure	Last blood pressure measurement <140/85 mmHg in the preceding 12 months.
Process QI	HbA1c	At least one measurement of HbA1c in the preceding 12 months.
**Non-incentivized QIs**		
Process QI	Blood pressure	At least one blood pressure measurement in the preceding 12 months.
Clinical QI	HbA1c	HbA1c level <7.5% in the preceding 12 months.
Process QI	Cholesterol	At least one cholesterol measurement in the preceding 12 months.
Clinical QI	Cholesterol	Total cholesterol <5 mmol/l in the preceding 12 months.

*QI, quality indicator*.

### Outcomes

Primary outcome: Between-group differences in the proportions of patients meeting incentivized QIs after 24 months (Table 1).

Secondary outcome: Between-group differences in the proportions of patients meeting non-incentivized QIs after 24 months (Table 1).

We assessed the proportions of patients meeting QIs at baseline and every 2 months until the trial was terminated after 24 months.

### Randomization and Sample Size

We cluster-randomized at the practice level to minimize contamination between the groups. Randomization was constrained by the proportion of patients meeting clinical QIs at baseline per practice, the number of participating GPs per practice, GP network participation, and the number of patients with diabetes mellitus per practice. More details on the randomization and the sample size calculation can be found in Meier et al. (8).

### Sensitivity Analysis

We also assessed the performance of GPs who were part of the FIRE project but had refused to participate in the randomized controlled trial. The analysis was carried out retrospectively for the same time points as in the randomized controlled trial. With this analysis, we aimed to investigate whether independent changes in the proportion of patients meeting the QIs occurred and whether receiving educational feedback alone had any effect.

### Statistical Methods

We reported categorical data as frequencies and percentages, continuous variables as means with SDs, or medians with interquartile ranges (IQRs), as appropriate. To assess the effect of the financial incentives, we used hierarchical multivariable logistic regression models, with practice and GPs nested within practices as random variables. Meeting a QI was the (Boolean) dependent variable, and independent variables were time and group allocation. To study whether the intervention effect varied over time, we added an interaction term between time and intervention to the model. Furthermore, we adjusted for the age and gender of the GPs and the number of patients with diabetes mellitus per GP. The same model was used for the sensitivity analysis, with the non-participating GPs as a third group allocation. To visualize trend and effect, we computed each GP's proportion of patients meeting the QIs, aggregated within group and time point, and computed means and the Wald-95% CIs.

## Results

### Study Population

A total of 71 GPs gave consent to participate in our study. Randomization allocated 21 practices with 36 GPs to the intervention group and 22 practices with 35 GPs to the control group. Subsequently, all patients with diabetes mellitus were included, resulting in an intention to treat a population of 3,854 patients (the intervention group: 1,786 patients and the control group: 2,068 patients). In the 12 months intervention period, four GPs dropped out of the intervention group (163 patients) and two GPs dropped out of the control group (176 patients). Throughout the 12 months of follow-up, another four GPs dropped out of the intervention group (138 patients) and five GPs dropped out of the control group (309 patients) (Figure 1).

Information on reasons and dates of dropouts of GPs is available in Supplementary Material 1 and Table 1. The patient fluctuation was caused by death, changes in eligibility status (no consultation within 12 months), or newly identified patients with diabetes mellitus. Of the initially included 3,189 patients, 2,742 (71.1%) were observed over the entire study period (Figure 1). The study ended as planned after 24 months.

At baseline, GPs had a median age of 52 years (IQR: 44–60), 72% were male and 91.5% worked in a group practice. The patients had a median age of 70 years (IQR: 60–78), 57% were male. Detailed information on the baseline characteristics of the study population is provided in Meier et al. (8). The numbers of measurements of BP, HbA1c, and cholesterol and also the respective clinical values at baseline and in the two study phases are given in Table 2.

**Table 2:** Numbers of measurements per patient, and clinical values of BP, HbA1c, and cholesterol over the entire study period.

	**Group**	**Baseline 12 months**	**Intervention phase 12 months**	**Follow-up phase 12 months**
BP measures [median (IQR)]	Intervention	3 [1–4]	3 [2–4]	3 [2–4]
	Control	2 [1–4]	2 [1–4]	2 [1–4]
Systolic BP [mmHg] [median (IQR)]	Intervention	137.5 [128.5–149.0]	136.8 [127.7–146.7]	135.6 [127.7–147.0]
	Control	134.0 [125.0–143.3]	134.0 [124.5–143.3]	135.0 [125.1–145.0]
Diastolic BP [mmHg] [median (IQR)]	Intervention	80.0 [73.9–86.0]	80.0 [73.6–85.0]	79.2 [73.3–85.0]
	Control	80.0 [73.3–85.0]	79.0 [73.0–85.0]	80.0 [74.0–85.0]
HbA1c measures [median (IQR)]	Intervention	2 [1–3]	2 [1–3]	2 [1–3]
	Control	3 [1–4]	2 [1–3]	2 [1–4]
HbA1c [%] [median (IQR)]	Intervention	6.8 [6.3–7.5]	6.8 [6.3–7.5]	6.9 [6.3–7.6]
	Control	6.8 [6.3–7.5]	6.8 [6.2–7.5]	6.8 [6.2–7.5]
Cholesterol measures [median (IQR)]	Intervention	1 [1–2]	1 [1–1]	1 [1–2]
	Control	1 [1–1]	1 [1–1]	1 [1–1]
Cholesterol [mmol/l] [median (IQR)]	Intervention	4.5 [3.8–5.3]	4.3 [3.7–5.4]	4.3 [3.6–5.2]
	Control	4.6 [3.8–5.4]	4.6 [3.8–5.4]	4.4 [3.7–5.2]

*BP, blood pressure; IQR, interquartile range*.

The median incentive paid to the GPs in the intervention group was 637.50 Swiss francs (IQR: 300–1,200) (€: 603.0, IQR: 284–1,137).

### Primary Outcomes

**Figure 2:**
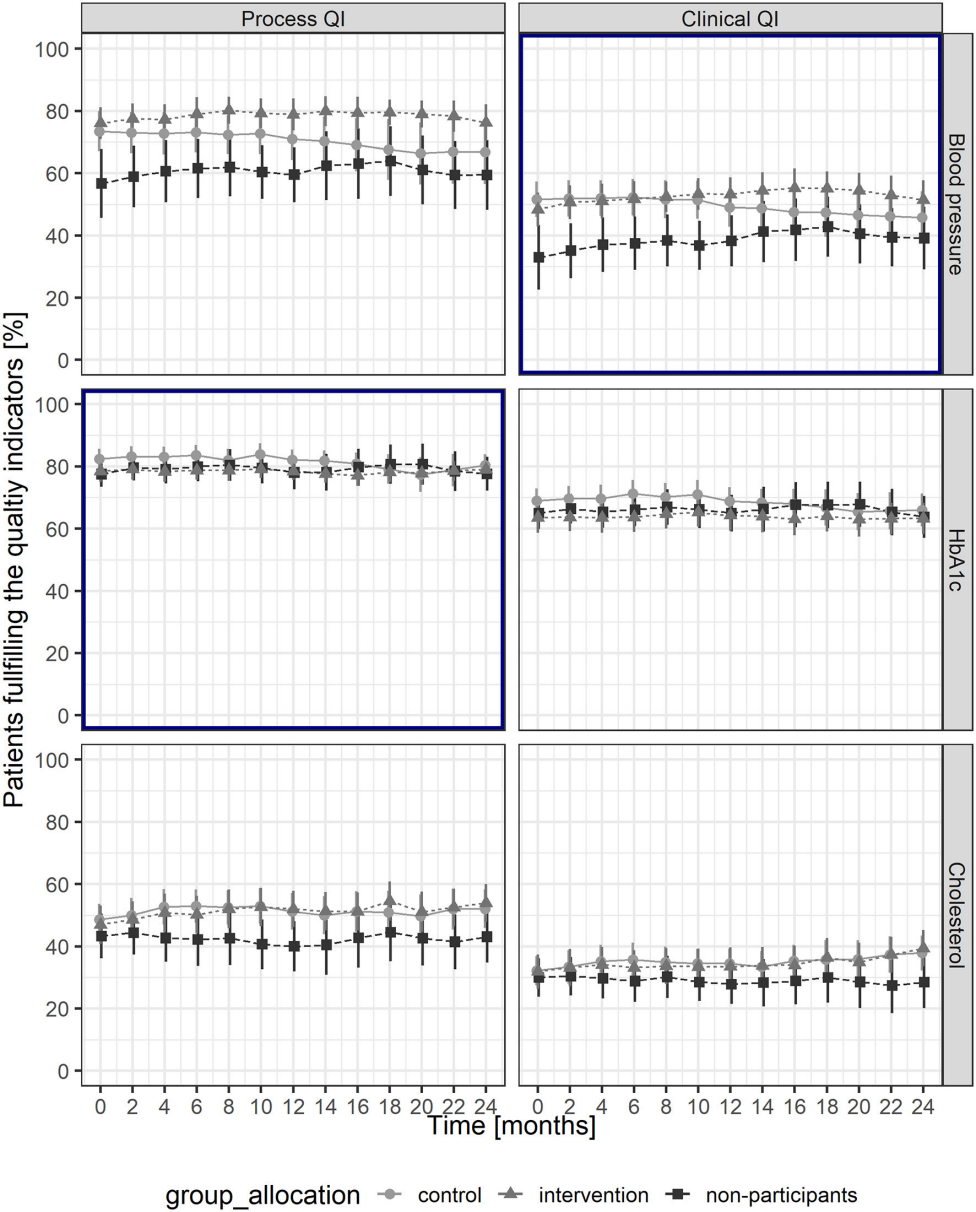
Proportions of patients fulfilling the quality indicators throughout the study period, with mean and Wald-95% CI for each group. Bold frames indicate primary outcomes. QI, quality indicator.

Throughout the study, the proportion for the process QI HbA1c was stable in the intervention group (intervention phase: 78.8–78.1%; follow-up phase 78.1–78.9%) and decreased slightly in the control group (intervention phase: 81.5–81.9%; follow-up phase 81.9–80.2%). The proportion of patients achieving a blood pressure target levels below 140/85 mmHg increased in the intervention group (intervention phase: 49.7–52.5%; follow-up phase 52.5–51.9%) and decreased in the control group (intervention phase: 51.2–48.9%; follow-up phase 48.9–47.2%). For illustration, see Figure 2.

The odds ratio (OR) for the interactive effect of time and intervention over the entire study period was 1.21 (95% CI: 1.04–1.42, *p* < 0.05) for the process QI of HbA1c and 1.18 (95% CI: 1.04–1.35, *p* < 0.05) for the clinical QI of achieving a blood pressure target level below 140/85 mmHg (Table 3). The detailed results of the logistic regression and the estimated random effects of GPs and GPs nested in practices can be found in Supplementary Material 1; Tables 2, 3.

**Table 3:** Interactive effect of time and intervention over the entire study period of 24 months.

**Type**	**Subject**	**OR**	**95% CI**	***p*-value**
**Primary outcomes**				
Clinical QI	Blood pressure	1.18	1.04–1.35	<0.05
Process QI	HbA1c	1.21	1.04–1.42	<0.05
**Secondary outcomes**				
Process QI	Blood pressure	1.14	0.97–1.34	0.11
Clinical QI	HbA1c	1.11	0.97–1.27	0.13
Process QI	Cholesterol	1.21	1.06–1.38	<0.01
Clinical QI	Cholesterol	1.10	0.96–1.26	0.17

*The model is adjusted for the age and gender of the GPs and the number of patients with diabetes mellitus per GP*.

*QI, quality indicator; OR, odds ratio*.

### Secondary Outcomes

After 24 months, the proportions of patients meeting the cholesterol process QI and the clinical QIs increased in the intervention and the control groups, the corresponding proportion for the HbA1c process QI was stable, whereas the proportion for the BP process QI decreased in the control group (Figure 2). The logistic regression analysis revealed a significant effect of financial incentives on the cholesterol process QI (OR: 1.21; 95% CI: 1.06–1.38, *p* > 0.01) (Table 3).

### Sensitivity Analysis

For the sensitivity analysis, we included 25 GPs from 15 different practices with 1,341 patients with diabetes mellitus. Practice, GP, and patient characteristics were similar to the study population, whereas baseline proportions of patients meeting QIs were lower for non-participants regarding blood pressure and cholesterol QIs (Figure 2). The results of the logistic regressions showed that no factors besides the intervention did affect the outcomes during the study period (Supplementary Material 1 and Supplementary Table 4) and that the educational feedback report had no effect.

## Discussion

In this follow-up analysis of our cluster randomized parallel controlled trial, we evaluated the long-term effect of financial incentives on treatment quality of patients with diabetes mellitus for 12 months beyond incentives were stopped, compared to educational feedback reports. Compared to the baseline levels, the intervention group achieved an improvement of 2% in the proportion of patients achieving the recommended blood pressure target levels, whereas the proportion of patients receiving annual HbA1c measurements remained stable.

The most relevant and significant effect of financial incentives could be observed on the proportion of patients achieving the recommended blood pressure target levels. In the intervention group, the proportion increased steadily from the beginning of the trial until 18 months after the start of the study (6 months after the financial incentives were stopped). During this period, a significant increase of about 5% was achieved, corresponding to a decrease in the median systolic blood pressure of about 2 mmHg. After this peak performance, the proportion decreased by ~3% in the last 6 months of the study. In the control group, the proportion fell 3% over the entire study period.

Interestingly, and in contrast to the analysis performed directly after the intervention phase (8), we could now observe an effect of financial incentives. Surprising, although by no means inexplicable, was the delay in peak performance until 6 months after the intervention ended. A time window of 12 months seemed to be too short to observe a consistent reduction in blood pressure in all practices. Even though patients with diabetes mellitus are a frequently visiting patient cohort, it takes a certain amount of time before they are seen for their regular control consultations and changes in hypertension treatment show.

The decline in the last 6 months of the follow-up phase reflects an essential challenge in implementing financial incentives, namely, the lack of sustainability of their effects. As other studies reported, a drop in performance after withdrawal of the incentives is common and can even reach pre-incentive levels (3–5). The mechanisms behind this decline following the removal of financial incentives are not yet fully elucidated. A potential explanation is that financial incentives, as external motivators, may crowd out intrinsic motivation; however, particularly in studies assessing health workers, the results are conflicting (13, 14).

The proportions of patients receiving annual HbA1c measurements were stable at ~80% in both the intervention and control groups. However, the logistic regression, which took the interactive effect of time and intervention into account, revealed a significant difference between the intervention and control groups. We attribute this difference to a decrease in performance in the control group during the follow-up period, partially explainable by the COVID-19 pandemic in spring 2020 (around month 20 in the study period). For several weeks, GPs in Switzerland were only allowed to hold emergency consultations, which might have negatively impacted the care of patients with diabetes (15). Why this should affect only certain practices in the control group we cannot explain; however, shortly after the restrictions were lifted, previous levels were swiftly reached again in these affected practices. The lack of improvement in meeting the process QI of measuring HbA1c, for which rates of 80% were achieved, may be explained by a ceiling effect. This hypothesis gets support from our logistic regression models, which reveal a smaller variability of random effects on practice and GP levels for the QIs addressing HbA1c than for the other QIs. The assumption that a ceiling effect prevented further improvement above 80% is further supported by the fact that it is generally easier to improve process QIs than clinical QIs (16, 17).

In contrast to the results of the analyses immediately after the intervention phase, the spill-over effect of financial incentives on the process QI of measuring blood pressure values vanished (8). The effect on the process QI of measuring cholesterol, however, persisted. The evidence regarding spill-over effects on non-incentivized QIs and reporting of risk factors is inconclusive (18–21), and with care quality, in the control group and among the non-participants improving slightly we cannot fully exclude any study-independent effects.

### Strengths and Limitations of This Study

To our knowledge, we conducted the first randomized controlled trial testing financial incentives for improved diabetes treatment in Europe. In general, studies to improve the quality of diabetes care are of high importance because of the high prevalence and burden of disease. We were able to implement a simple but effective intervention and blind the control group. In addition, we were able to compare the study results with another cohort not participating in the study. This study thus closes a gap, left by observational studies. Moreover, the baseline analysis showed that the patient population in this trial was highly comparable to other diabetes populations in Swiss primary care (22–24).

The EMR database from which the study draws may represent a potential limitation since such databases are known to be prone to missing data and data quality issues. These issues might not be apparent at baseline due to randomization. However, we cannot preclude that the increases in proportions of patients meeting process QIs are due to better data reporting rather than improved quality of care. This confounder is of particular concern in the case of the process QI of blood pressure measurement, as it is very likely that blood pressure values are not always reported in the dedicated field of the entry mask. However, these issues do not necessarily affect improvements in clinical QIs. A potential limitation is the risk of (self-) selection bias due to specific GPs participating or dropping out during the study. However, a dedicated subgroup analysis showed that GPs dropping out during the study were not different in performance from GPs remaining in the study. At last, information about the death of a patient is often missing in the FIRE database, since few GPs code it appropriately. To counteract this limitation, patients with no consultation within the last 12 months were excluded as losses of follow-up.

## Conclusion

After the withdrawal of financial incentives for the GPs after 12 months, some QIs still improved, suggesting that 1 year might be too short to observe the full effect of such interventions. The decrease in QI achievement rates after 18 months suggests that the positive effects of time-limited financial incentives eventually wane.

## Data Availability Statement

The data are gathered within the ongoing FIRE project. The FIRE database can be accessed at any time by the scientific team of the institute. For external requests, access has to be requested from the head of the institute.

## Ethics Statement

The local Ethics Committee of the Canton of Zurich waived approval, because the project is outside the scope of the law on human research (BASEC-Nr. Req-2017-00797).

## Author Contributions

RM: methodology, formal analysis, writing—original draft, review, and editing. CC: conceptualization, methodology, writing—review and editing, and funding acquisition. FV: data curation, formal analysis, methodology, and writing—review and editing. LM: methodology and writing—review and editing. OS and TR: conceptualization, writing—review and editing, and funding acquisition. All authors contributed to the article and approved the submitted version.

## Funding

This study was supported by a grant from the Swiss National Science Foundation, Grant number 407440_167204.

## Conflict of Interest

The authors declare that the research was conducted in the absence of any commercial or financial relationships that could be construed as a potential conflict of interest.

## Publisher's Note

All claims expressed in this article are solely those of the authors and do not necessarily represent those of their affiliated organizations, or those of the publisher, the editors and the reviewers. Any product that may be evaluated in this article, or claim that may be made by its manufacturer, is not guaranteed or endorsed by the publisher.
