# Supplementary material for: Long-Term Effects of Financial Incentives for General Practitioners on Quality Indicators in the Treatment of Patients With Diabetes Mellitus in Primary Care—A Follow-Up Analysis of a Cluster Randomized Parallel Controlled Trial
Source: Front Med (Lausanne). 2021 Oct 26;8:664510. doi: 10.3389/fmed.2021.664510 (PMC8576070; doi:10.3389/fmed.2021.664510)
Supplement: Supplementary file 1 [file Data_Sheet_1.docx]

Example of an educational diabetes feedback report


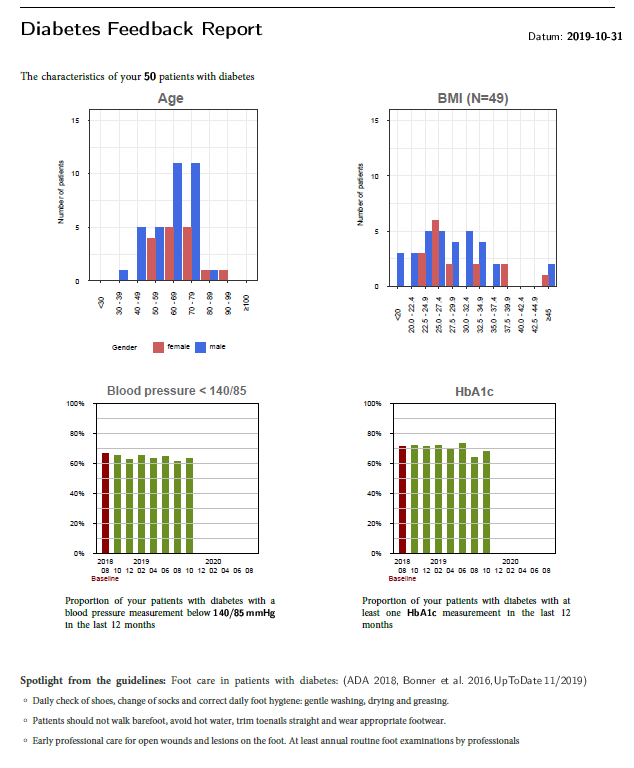


Supplementary table 1 Reason for dropout and length of observation period of GPs dropping out:

|  | **period** | **Reason for dropout** | **Length of observation period** | **Number of patients dropping out** |
| --- | --- | --- | --- | --- |
| **Intervention group** | intervention | GP left the practice | 6 month observation period | 33 |
|  | intervention | GP left the practice | 8 month observation period | 42 |
|  | intervention | Data transmission problems | 2 month observation period | 52 |
|  | intervention | Data transmission problems | 2 month observation period | 34 |
|  | follow-up | GP retired | 16 month observation period | 34 |
|  | follow-up | loss to follow up | 14 month observation period | 45 |
|  | follow-up | GP maternity leave | 18 month observation period | 17 |
|  | follow-up | GP left the practice | 12 month observation period | 42 |
| **Control group** | intervention | GP left the practice | 8 month observation period | 55 |
|  | intervention | GP retiered | 2 month observation period | 121 |
|  | follow-up | data transfer | 12 month observation period | 26 |
|  | follow-up | loss to follow up | 22 month observation period | 33 |
|  | follow-up | left the practice | 16 month observation period | 79 |
|  | follow-up | left the practice | 12 month observation period | 112 |
|  | follow-up | left the practice | 12 month observation period | 59 |

Supplementary table 2: Detailed results of logistic regression models for primary and secondary outcomes over the entire study period

|  | **Type** | **Subject** | **Variable** | **OR** | **95% CI** | **p - value** | |
| --- | --- | --- | --- | --- | --- | --- | --- |
| **Primary outcomes** | Clinical QI | BP | GP gender (ref = female) | 0.01 | 0.00 - 0.63 | | <0.05 |
|  |  |  | GP age | 1.18 | 0.95 - 1.47 | | 0.138 |
|  |  |  | Volume of patients with diabetes | 1.83 | 0.6 - 5.64 | | 0.289 |
|  |  |  | Main effect intervention | 0.42 | 0.00 - 50.22 | | 0.721 |
|  |  |  | Main effect time | 0.83 | 0.76 - 0.91 | | <0.001 |
|  |  |  | Interactive effect time*intervention | 1.18 | 1.04 - 1.35 | | <0.05 |
|  | Process QI | HbA1c | GP gender (ref = female) | 0.11 | 0.00 - 3.40 | | 0.206 |
|  |  |  | GP age | 0.87 | 0.73 - 1.03 | | 0.096 |
|  |  |  | Volume of patients with diabetes | 1.63 | 0.68 - 3.94 | | 0.275 |
|  |  |  | Main effect intervention | 0.04 | 0.00 - 1.71 | | 0.092 |
|  |  |  | Main effect time | 0.77 | 0.69 - 0.86 | | <0.001 |
|  |  |  | Interactive effect time*intervention | 1.21 | 1.04 - 1.42 | | <0.05 |
| **Secondary outcomes** | Process QI | BP | GP gender (ref = female) | 0.00 | 0.00 - 1.53 | | 0.069 |
|  |  |  | GP age | 1.23 | 0.93 - 1.62 | | 0.156 |
|  |  |  | Volume of patients with diabetes | 2.50 | 0.51 - 12.32 | | 0.26 |
|  |  |  | Main effect intervention | 9.96 | 0.01 - 6898.64 | | 0.491 |
|  |  |  | Main effect time | 0.72 | 0.65 - 0.80 | | <0.001 |
|  |  |  | Interactive effect time*intervention | 1.14 | 0.97 - 1.34 | | 0.113 |
|  | Clinical QI | HbA1c | GP gender (ref = female) | 0.06 | 0.00 - 1.95 | | 0.113 |
|  |  |  | GP age | 0.92 | 0.78 - 1.10 | | 0.366 |
|  |  |  | Volume of patients with diabetes | 2.10 | 0.85 - 5.18 | | 0.107 |
|  |  |  | Main effect intervention | 0.07 | 0.00 - 4.57 | | 0.214 |
|  |  |  | Main effect time | 0.87 | 0.79 - 0.96 | | <0.01 |
|  |  |  | Interactive effect time*intervention | 1.11 | 0.97 - 1.27 | | 0.128 |
|  | Process QI | Chol | GP gender (ref = female) | 0.08 | 0.00 - 8.25 | | 0.289 |
|  |  |  | GP age | 0.86 | 0.69 - 1.08 | | 0.193 |
|  |  |  | Volume of patients with diabetes | 2.14 | 0.69 - 6.63 | | 0.189 |
|  |  |  | Main effect intervention | 0.13 | 0.00 - 19.07 | | 0.426 |
|  |  |  | Main effect time | 0.91 | 0.83 - 0.99 | | <0.05 |
|  |  |  | Interactive effect time*intervention | 1.21 | 1.06 - 1.38 | | <0.01 |
|  | Clinical QI | Chol | GP gender (ref = female) | 0.17 | 0.00 - 13.32 | | 0.426 |
|  |  |  | GP age | 0.84 | 0.68 - 1.04 | | 0.104 |
|  |  |  | Volume of patients with diabetes | 2.21 | 0.70 - 6.94 | | 0.175 |
|  |  |  | Main effect intervention | 0.20 | 0.00 - 39.42 | | 0.549 |
|  |  |  | Main effect time | 1.05 | 0.96 - 1.15 | | 0.284 |
|  |  |  | Interactive effect time*intervention | 1.10 | 0.96 - 1.26 | | 0.171 |

Supplementary table 3: Random effects of hierarchical logistic model

| **QI** | **Cluster** | **Variance** | **Std. deviation** |
| --- | --- | --- | --- |
| bp_proc_qual | arzt_id:praxis_id | 0.47 | 0.68 |
| bp_proc_qual | praxis_id | 0.39 | 0.62 |
| bp_out_qual | arzt_id:praxis_id | 0.31 | 0.56 |
| bp_out_qual | praxis_id | 0.17 | 0.41 |
| hba1c_proc_qual | arzt_id:praxis_id | 0.16 | 0.39 |
| hba1c_proc_qual | praxis_id | 0.12 | 0.35 |
| hba1c_out_qual | arzt_id:praxis_id | 0.14 | 0.38 |
| hba1c_out_qual | praxis_id | 0.20 | 0.44 |
| chol_proc_qual | arzt_id:praxis_id | 0.32 | 0.57 |
| chol_proc_qual | praxis_id | 0.21 | 0.45 |
| chol_out_qual | arzt_id:praxis_id | 0.23 | 0.48 |
| chol_out_qual | praxis_id | 0.33 | 0.57 |

Supplementary table 4: Detailed results of logistic regression models for primary and secondary outcomes for the additional analysis

|  | **Type** | **Subject** | **Variable** | **OR** | **95% CI** | **p-value** |
| --- | --- | --- | --- | --- | --- | --- |
| **Primary outcomes** | Clinical QI | BP | GP gender (ref = female) | 0.77 | 0.55 - 1.09 | 0.147 |
|  |  |  | GP age | 1.01 | 0.99 - 1.03 | 0.184 |
|  |  |  | Volume of patients with diabetes | 1.04 | 0.96 - 1.13 | 0.3 |
|  |  |  | Main effect intervention «non-participants» | 0.51 | 0.31 - 0.85 | <0.05 |
|  |  |  | Main effect intervention «treat» | 0.92 | 0.58 - 1.47 | 0.739 |
|  |  |  | Main effect time | 0.98 | 0.98 - 0.99 | <0.001 |
|  |  |  | Interactive effect time*intervention «non-participants» | 1.01 | 1.00- 1.02 | 0.081 |
|  |  |  | Interactive effect time*intervention «treat» | 1.01 | 1.00- 1.03 | <0.05 |
|  | Process QI | HbA1c | GP gender (ref = female) | 0.80 | 0.62 - 1.02 | 0.071 |
|  |  |  | GP age | 0.99 | 0.97 – 1.00 | 0.056 |
|  |  |  | Volume of patients with diabetes | 1.05 | 0.99 - 1.11 | 0.123 |
|  |  |  | Main effect intervention «non-participants» | 0.74 | 0.52 - 1.06 | 0.097 |
|  |  |  | Main effect intervention «treat» | 0.75 | 0.55 - 1.04 | 0.081 |
|  |  |  | Main effect time | 0.98 | 0.97 - 0.99 | <0.001 |
|  |  |  | Interactive effect time*intervention «non-participants» | 1.01 | 1.00- 1.03 | 0.071 |
|  |  |  | Interactive effect time*intervention «treat» | 1.02 | 1.00- 1.03 | <0.05 |
| **Secondary outcomes** | Process QI | BP | GP gender (ref = female) | 0.79 | 0.52 - 1.19 | 0.26 |
|  |  |  | GP age | 1.02 | 0.99 - 1.04 | 0.151 |
|  |  |  | Volume of patients with diabetes | 1.05 | 0.95 - 1.16 | 0.314 |
|  |  |  | Main effect intervention «non-participants» | 0.46 | 0.25 - 0.86 | <0.05 |
|  |  |  | Main effect intervention «treat» | 1.23 | 0.70 - 2.17 | 0.47 |
|  |  |  | Main effect time | 0.97 | 0.96 - 0.98 | <0.001 |
|  |  |  | Interactive effect time*intervention «non-participants» | 1.02 | 1.01- 1.03 | <0.01 |
|  |  |  | Interactive effect time*intervention «treat» | 1.01 | 1.00- 1.02 | 0.113 |
|  | Clinical QI | HbA1c | GP gender (ref = female) | 0.80 | 0.62 - 1.04 | 0.091 |
|  |  |  | GP age | 0.99 | 0.98 - 1.00 | 0.159 |
|  |  |  | Volume of patients with diabetes | 1.07 | 1.01 - 1.13 | <0.05 |
|  |  |  | Main effect intervention «non-participants» | 0.75 | 0.53 - 1.07 | 0.114 |
|  |  |  | Main effect intervention «treat» | 0.79 | 0.57 - 1.09 | 0.147 |
|  |  |  | Main effect time | 0.99 | 0.98 - 1.00 | <0.01 |
|  |  |  | Interactive effect time*intervention «non-participants» | 1.01 | 1.00- 1.03 | <0.05 |
|  |  |  | Interactive effect time*intervention «treat» | 1.01 | 1.00- 1.02 | 0.128 |
|  | Process QI | Chol | GP gender (ref = female) | 0.82 | 0.59 - 1.15 | 0.251 |
|  |  |  | GP age | 0.99 | 0.97 - 1.01 | 0.342 |
|  |  |  | Volume of patients with diabetes | 1.05 | 0.97 - 1.13 | 0.228 |
|  |  |  | Main effect intervention «non-participants» | 0.60 | 0.38 - 0.95 | <0.05 |
|  |  |  | Main effect intervention «treat» | 0.85 | 0.57 - 1.28 | 0.445 |
|  |  |  | Main effect time | 0.99 | 0.98 - 1.00 | <0.05 |
|  |  |  | Interactive effect time*intervention «non-participants» | 1.00 | 0.99 - 1.01 | 0.996 |
|  |  |  | Interactive effect time*intervention «treat» | 1.02 | 1.00- 1.03 | <0.01 |
|  | Clinical QI | Chol | GP gender (ref = female) | 0.81 | 0.59 - 1.12 | 0.209 |
|  |  |  | GP age | 0.99 | 0.97 - 1.00 | 0.111 |
|  |  |  | Volume of patients with diabetes | 1.06 | 0.99 - 1.14 | 0.12 |
|  |  |  | Main effect intervention «non-participants» | 0.69 | 0.43 - 1.11 | 0.127 |
|  |  |  | Main effect intervention «treat» | 0.86 | 0.56 - 1.33 | 0.507 |
|  |  |  | Main effect time | 1.00 | 1.00- 1.01 | 0.28 |
|  |  |  | Interactive effect time*intervention «non-participants» | 0.99 | 0.98 - 1.00 | 0.152 |
|  |  |  | Interactive effect time*intervention «treat» | 1.01 | 1.00- 1.02 | 0.169 |

OR: odds ratio; CI: confidence interval; QI: quality indicator; BP: blood pressure; Chol: cholesterin; GP: General practitioner
